# Hybridization and introgression between two fig trees with contrasting ecological preferences

**DOI:** 10.1186/s12862-025-02476-7

**Published:** 2025-12-24

**Authors:** Ramil Kohkaew, Yamei Ding, Pornwiwan Pothasin, Nattaya Srisawad, Stephen G. Compton, Hui Yu

**Affiliations:** 1 State Key Laboratory of Plant Diversity and Specialty Crops, South China Botanical Garden, Chinese Academy of Sciences, Guangzhou, Guangdong 510650 China; 2 University of Chinese Academy of Sciences, Beijing, 100049 China; 3 Conservation Biology Program, School of Interdisciplinary, Mahidol University, Kanchanaburi Campus, Kanchanaburi, 71150 Thailand; 4 Insititute of Molecular Biosciences, Mahidol University, Nakhon Pathom, 73170 Thailand; 5 School of Biology, University of Leeds, Leeds, LS2 9JT UK; 6 Guangdong Provincial Key Laboratory of Applied Botany, South China Botanical Garden, Chinese Academy of Sciences, Guangzhou, Guangdong 510650 China

**Keywords:** *Ficus hispida*, *Ficus squamosa*, Hybridization, Leaf traits, Riparian area

## Abstract

Hybridization in plants with specialized pollination systems provides insights into the mechanisms of species boundary maintenance and breakdown. The significance of hybridization in the history of ecologically important fig trees is unclear. *Ficus hispida* and *Ficus squamosa* are dioecious species with contrasting habitats and dispersal mechanisms. We combined phenological, morphological and genomic analyses to characterize putative hybrids and interspecific gene flow. Overlapping fruiting periods during Thailand’s rainy season potentially allow reciprocal movement of pollinators between the two species. Putative hybrid individuals displayed intermediate morphological traits but had larger leaves. Genomic analyses using single nucleotide polymorphisms confirmed the hybrid status of individuals identified by their morphology. They also revealed patterns of genetic admixture with parent species. Phylogenetic analysis of chloroplast genomes also suggested historical introgression events between the two species. The hybrids were generally found in riparian areas habitats required by *F. squamosa*, where environmental conditions may be intermediate with the drier open conditions favored by *F. hispida*. Fig tree pollination depends on fig wasps that are generally host-species specific, but clearly some host switching occurs. *Ficus squamosa* has highly modified seeds adapted for dispersal by water, whereas *F. hispida* depends on seed dispersal by vertebrates. Aquatic-dispersal modifications are poorly developed in the hybrids, suggesting they require seed dispersal by frugivores. This study provides evidence of hybridization between two fig tree species with contrasting ecological preferences, but the evolutionary consequences of the inter-specific pollen-flow remain uncertain.

**Supplementary Information:**

The online version contains supplementary material available at 10.1186/s12862-025-02476-7.

## Introduction

The significance of hybridization in plant evolution and speciation is increasingly recognised, with hybridization seen as playing a fundamental role by contributing to adaptation and biodiversity. Hybridization is the process of interbreeding between individuals from two distinct populations, varieties, species, or genera, resulting in hybrid offspring. This definition assumes that the parents are genetically differentiated to the point where they would not typically mate in nature, or if they do, their offspring face some challenges (like infertility) [1–6]. Here we use hybridization to mean interbreeding between two genetically differentiated taxathat produces hybrid offspring and permits interspecific gene flow. Outcomes of hybridization can range from generating sterile to fertile hybrids and subsequent introgression, and the strength of any reproductive barriers can vary among taxa and contexts. In this study, we test for genomic admixture consistent with hybridization; we do not assign hybrid classes (e.g., F_1_ vs. backcrosses).

Plants with specialized nursery pollination systems have undergone extensive diversification. This radiation is likely facilitated by a combination of factors, including the potential for hybridization and pollinator host shifts, as seen in the *Yucca*–*Tegeticula* [7], the Phyllanthaceae–*Epicephala* mutualism [8], and the *Ficus*-pollinating wasp mutualism [9]. These three systems are iconic because they are seen to represent extreme examples of coevolutionthat can demonstrate how inter-species relationships can drive the diversification of entire lineages, leading to mirrored evolutionary trees of plants and mutualists. Reflecting this, phylogenetic studies of these systems reveal that pollinator host shifts are often associated with a degree of parallel cospeciation between the insects and their hosts [10–12].

The intimate mutualism between fig trees (*Ficus* spp.) and their pollinating wasps represents one of the most remarkable examples of plant-insect coevolution in nature.

This relationship, in place for over 60 million years, was traditionally viewed as a highly specific, one-to-one interaction where each fig species is pollinated by a unique species of fig wasp [13–16]. This paradigm of strict specificity was fundamental to the natural history studies of Galil [14], Janzen [15] and Wiebes [16]. However, more recent molecular studies have revealed a more complex picture, confirming widespread co-diversification [12] but also documenting numerous exceptions to the one-to-one rule where host sharing and pollinator switching occur [13, 17]. Therefore, understanding the mechanisms that maintain species boundaries in a system where some exceptions occur is central to unraveling the evolutionary history of the mutualism. For instance, Wei et al. [17] and Yu et al. [18, 19] have shown that closely related sympatric *Ficus* species can maintain genetic distinctiveness even while sharing pollinator species, with gene flow inhibited by differences in fruiting phenologies [20].

An increasing number of *Ficus* species are known to support multiple pollinator fig wasp species across their geographical ranges. Examples range from *Ficus sur* in West Africa with two pollinator species [21] and *Ficus septica* in Southeast Asia with four [22], to *F. rubiginosa* in Australia with at least five [23] and *F. hirta* in SE Asia with nine [24]. Furthermore, widespread species like *F. racemosa* and *F. microcarpa* are also associated with multiple pollinators (at least eight for both species; J-Y. Rasplus, personal communication), as are sympatric closely related dioecious fig species in the Indo-Burma region such as, *F. auriculata*, *F. semicordata* and *F. oligodon* [25].

The examples above demonstrate intraspecific pollinator multiplicity, where a single fig species is associated with several specific pollinator species, often in different parts of a plant’s extensive range. A distinct, and potentially more consequential, complexity is interspecific pollinator sharing, where different fig species are pollinated by the same wasp species. This is exemplified by *F. hispida* and its relatives (*F. heterostyla*, *F. squamosa*, *F. treubii*), which form a *Ceratosolen* pollinator complex across their overlapping ranges [26, 27]. In such cases, pollinator host switches demonstrate that pre-zygotic isolation barriers can be permeable and can thereby lead to hybridization and introgression among the host fig species. Consequently, apparent co-diversification can be punctuated by some instances of host switching and hybridization events that challenge a simple co-evolutionary paradigm, and suggest a more complex and dynamic evolutionary history involving hybridization events and occasional pollinator sharing [12, 28–31]. Examples are providedby hybridization events in dioecious *Ficus* species on island populations in Indonesia and Japan [32, 33] and between two Australian fig species [34]. Hybridization appears to be more common in dioecious fig species [35]. It can significantly impact the fitness of both partners as shown by Ghana et al. [36] who demonstrated that hybridization between *F. montana* and *F. asperifolia* can disrupt the fig-pollinator mutualism, as seen in reduced development success of pollinator wasps in hybrid figs.

Recent studies of *F. squamosa*, a dioecious riparian species with modified seeds dispersed by water, have found that male figs of this species support development of at least two species of pollinators, one of which is more commonly present in figs of *F. hispida*, aclosely related open-ground species with seeds dispersed by birds and bats (P. Pothasin et al., unpublished). Putative hybrids between the two *Ficus* species were also identified suggesting that even though the growth forms, habitat preferences, adaptive strategies, and dispersal syndromes of these two species differ, hybridization events can occur where the species coexist. This evidence of hybridization among *Ficus* species has primarily been reported from botanical gardens and non-native ranges that may not reflect natural situations. Previous studies have documented that closely related sympatric *Ficus* species can suggest complementary fruiting phenologies and similar blends of attractant volatile organic compounds, are important for providing conditions that facilitate pollinator sharing and hybridization [19, 27, 37–40]. Furthermore, in two introduced Australian *Ficus* species (*F. aculeata* and *F. coronulata*), hybridization can produce individuals that mature successfully and lead to some backcrossing and introgression, though such hybrids are not common in nature [34].

The extreme ecological differences between their parent species make any hybrids between *F. squamosa* and *F. hispida* a particularly intriguing study system. We recorded the fruiting phenology of *F. squamosa* and compared it with that of *F. hispida* to check whether pollen flow was possible in both directions between these two species. We then identified putative hybrid individuals on the basis of their morphology and asked whether they exhibit intermediate or unique traits compared to the putative parent species. Using molecular methods, we then confirmed their hybrid status. For confirmed hybrids, we then determined whether they were primarily recent crosses (F₁s) or represented later-generation hybrids.

We examined the ecological background to the hybrid formation and its likely consequences by asking the following questions:1) Are the putative hybrids the result of hybridization between *F. hispida* and *F. squamosa*?; 2) Where are hybrids located in relation to habitat features such as proximity to water and the distribution of potential parent individuals?; 3) How is their morphology similar to or different from that of their parent species?

## Materials and methods

### Study sites and species

**Fig. 1:**
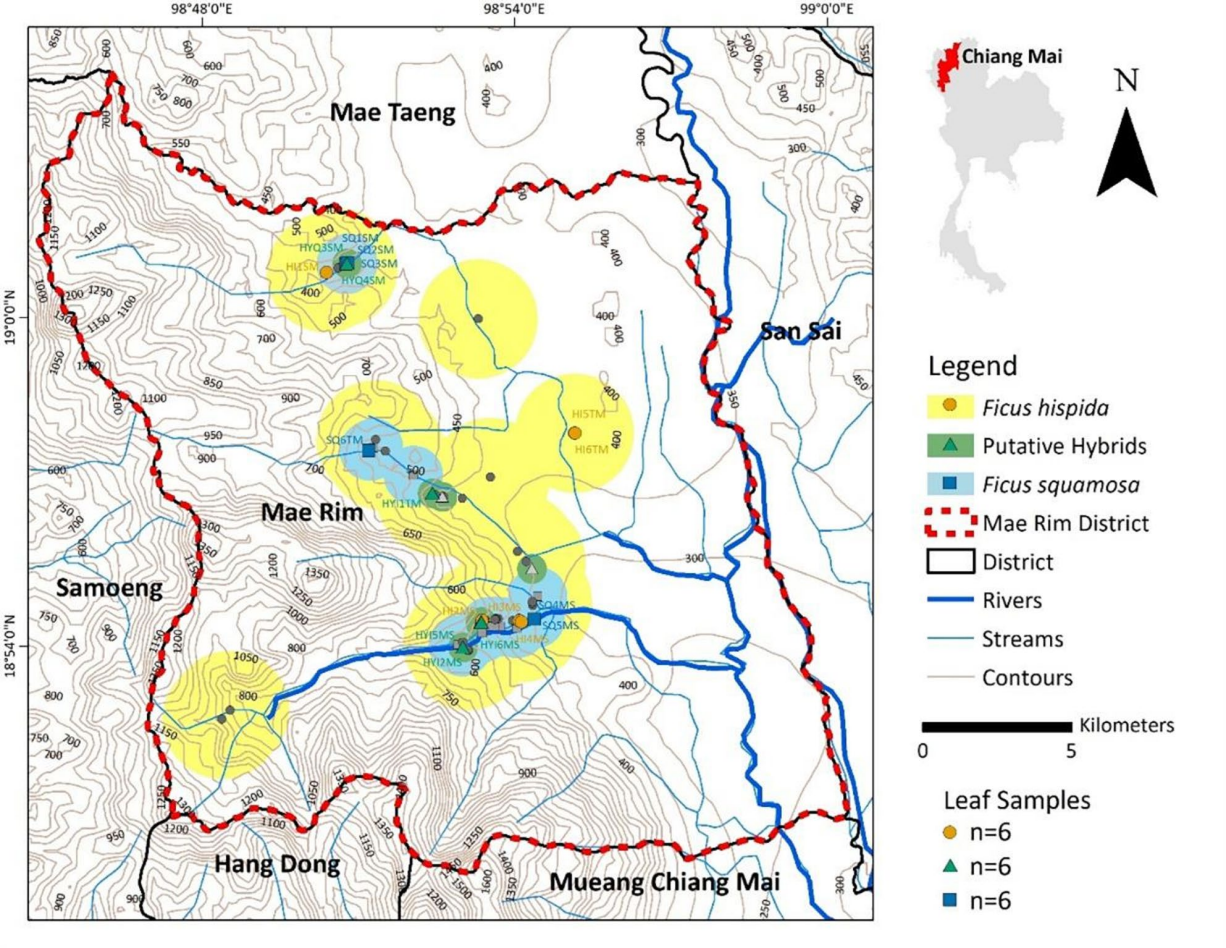
Distribution of the collected *Ficus hispida* (yellow circles), *F. squamosa* (blue squares), and putative hybrids (green triangles), with a total of 18 individuals (6 of each). Other records are shown in the same shapes but in gray. The circular area surrounding sample points indicates the buffer area, making the overlapping regions among the three taxa more visible

Field studies and plant sample collections were conducted in Mae Rim District, Chiang Mai Province, northern Thailand (18°54′50″N, 98°56′42″E) (Fig. 1) during the rainy season (June–September 2022). The region experiences a tropical monsoon climate with three distinct seasons: hot, rainy, and cool [41]. Temperatures range from highs of approximately 36 °C in the hot season to lows below 10 °C in mountainous areas during the cool season. Annual rainfall averages 1,200–1,500 millimetres, primarily falling between May and October [42]. Climate variability, driven by El Niño and La Niña phenomena, further influences temperature and rainfall patterns, while the mountainous terrain creates diverse microclimates that add to the region’s climatic complexity.

The plant materials, including putative natural hybrids analyzed in this study, were formally identified by Dr. Wattana Tanming, a taxonomist at the Queen Sirikit Botanic Garden (QSBG). Voucher specimens have been deposited at the South China Botanical Garden (SCBG) under the accession number SCBG20250825.

**Fig. 2:**
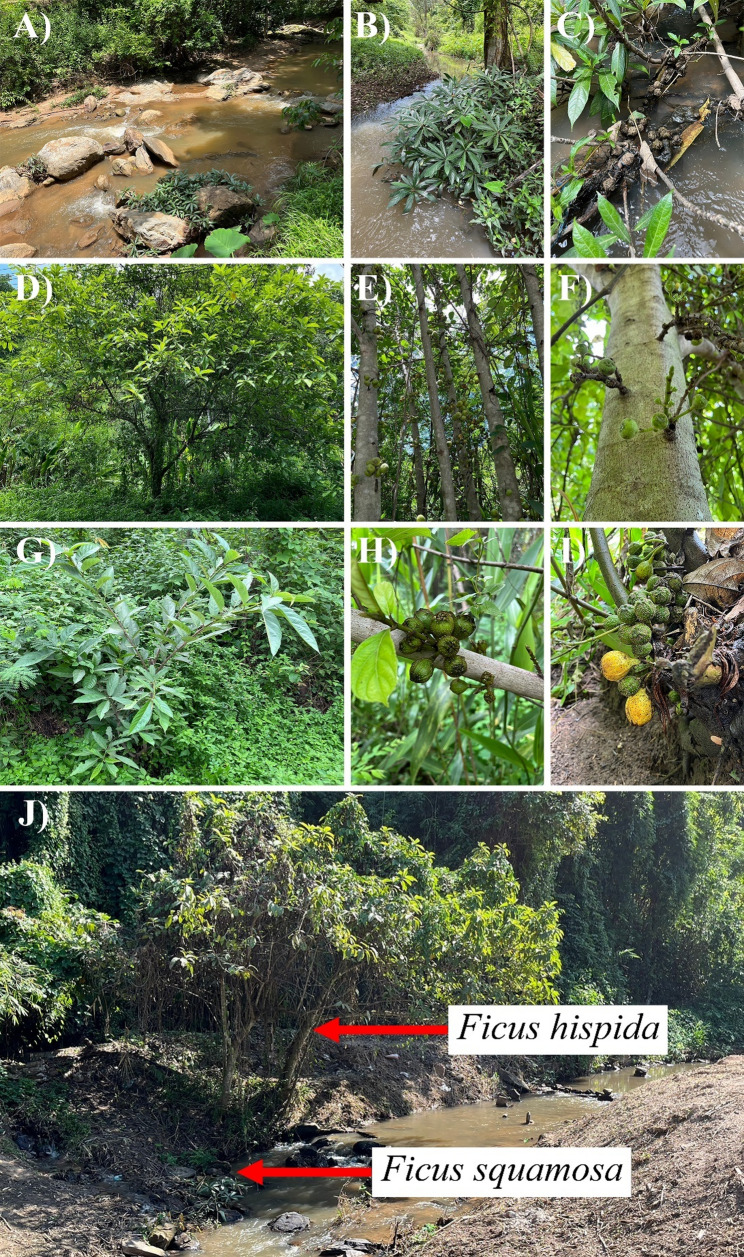
Species studied (**A**-**C**) *F. squamosa*, (**D**-**F**) *F. hispida*, (**G**-**I**) putative hybrids, and (**J**) *F. hispida* and *F. squamosa* in sympatric site

*Ficus squamosa* Roxb. (subgenus *Sycomorus*, section *Sycocarpus*) is a dioecious small riparian shrub with rooting stolon-like branchlets that grows approximately 1 to 2 m tall. It is a rheophyte, adapted to growing within or near fast-flowing rocky streams and at elevations up to 300–600 m. Its exceptional elongated styles may anchor waterborne fruits to river substrates, but by holding groups of seeds together they also form rafts that float on the water once the figs have broken open (Fig. 2). The natural distribution of *F. squamosa* covers Thailand through to Laos, Myanmar, China (Yunnan), NE India, Sikkim, Bhutan and Nepal, where it is mostly found in the rocky beds of quick running streams, and no more than 10 m from the water’s edge. Its figs are located along the branches and branchlets. The figs are rhombic-ovoid, reaching 3–4 cm in diameter at maturity, located along the branch and stolon-like roots near the ground. Both male and female figs remain yellow-green when ripe [9, 43–45].

*Ficus hispida* L. (subgenus *Sycomorus*, section *Sycocarpus*) is a dioecious coarsely hairy shrub or medium tree, up to 13 m tall, sometimes buttressed, cauliflorous or ramiflorous, leaves simple, opposite, sand-papery, figs are globose, up to 4 cm, clustered the main trunk and main branches, or borne in long clusters that can layon the ground at the base of the tree. The distribution of *F. hispida* covers South Asia (including India, Nepal, and Sri Lanka), South-east Asia, Malesia, Papua New Guinea and Australia (Queensland, Northern Territory and Western Australia). This species has wide ecological tolerances, being found in rainforest and gallery forest, open agricultural lands and river banks, up to 1200 m in altitude [9]. It is often found in human-disturbed and exposed areas in full sun. Its figs are located along the trunk and branch (Fig. 2). Both male and female figs are globose and turn yellow when ripe. They are possibly dispersed by birds, bats and arboreal mammals [46].

### Genomic analyses

Young leaves were collected from six individuals each of *F. hispida*, *F. squamosa*, and their putative hybrids. Leaf tissue was cut into pieces not exceeding 2 cm² and immediately dried using silica gel for DNA preservation [47]. For each sample, leaves were placed in a sealed bag with a minimum 10:1 ratio (silica gel: leaf mass) to ensure rapid desiccation. After 12 h, samples were checked for complete dryness by confirming that leaf pieces snapped cleanly when bent. The dried leaf material was then stored in sealed bags until DNA extraction.

### Genome sequencing and SNP genotyping

We performed whole-genome resequencing on the 18 individuals. Leaf sample DNA was extracted with the Qiagen DNeasy Plant Kit. Sequencing was conducted on Illumina platforms (HiSeq X Ten), yielding paired-end reads with a target coverage of 30×.

For raw resequencing reads, we used Trimmomatic v.0.39 [48] to trim low-quality bases. Bases with a quality lower than 20 were trimmed from the start or the end of reads, and the entire reads were discarded if shorter than 50 after trimming. Read quality was assessed with FastQC v.0.11.7 [49] before and after trimming. Remaining read pairs were mapped to a high-quality *F. hispida* reference genome [50] using BWA-MEM algorithm of BWA v.0.7.17 [51] with default parameters and sorted with SAMtools v.1.16 [52]. Then, MarkDuplicates tool from GATK v.4.2 [53] was used to mark PCR duplicates. Finally, we used GATK v.4.2 HaplotypeCaller to do per-sample variant detection and GenotypeGVCFs tool to perform joint genotyping. We further performed multiple filtering steps to only retain high-quality variants for downstream analysis.

To ensure high-quality SNPs and minimize genotype calling bias, we used a stepwise filtering pipeline. First, SNPs within 5 bp of an indel were removed. Subsequently, filtering was applied based on quality scores (QD < 2.0, MQ < 40, FS > 60, SOR > 3, MQRankSum < -12.5, ReadPosRankSum < -8). Additionally, non-biallelic SNPs and read depth (DP) lower than one third or higher than 2-fold of the average sequencing depth were removed. Furthermore, to obtain independent SNPs, we thinned the dataset at 5-kb intervals. Finally, singletons were removed to reduce false positives. The final dataset consisted of 12,046 independent SNPs for STRUCTURE, ADMIXTURE and Principal component analysis (PCA) analyses. We incorporated one *F. hirta* sample (https://ngdc.cncb.ac.cn/gsa/browse/CRA009554) as an outgroup and subjected it to the same filtering criteria, resulting in 7,837 high-quality SNPs for subsequent phylogenetic inference.

### Genetic clustering and admixture analysis

Population clustering analyses were performed based on STRUCTURE, ADMIXTURE, and PCA analysis. We used STRUCTURE v.2.3.4 [54] based on 12,046 SNPs to investigate the population structure across all individuals, with the number of clusters (K) being set from 1 to 4. To control for unequal sample sizes among species, we set POPALPHAS = 1 and initialized ALPHA to 0.25, following the recommendation by [55]. We assumed that allele frequencies were uncorrelated across populations. The analysis was conducted using 100,000 burn-in steps followed by 1,000,000 MCMC iterations, with 10 independent runs performed for each number of clusters (K) to assess the variability in log-likelihood values associated with each K. The optimal value of K was determined by parsimony index (PI) implemented in KFinder [56]. Furthermore, we also used ADMIXTURE v.1.3.0 [57] to infer population clustering with fivefold cross-validations for K from 1 to 4. An optimal K value was selected based on the lowest value of cross-validation error. PCA was run using the R package SNPRelate v. 1.6.2 [58] with default settings.

### Phylogeny analysis

A genetic distance matrix was calculated using VCF2Dis (https://github.com/BGI-shenzhen/VCF2D) based on 7,837 SNPs. Subsequently, a neighbor-joining (NJ) tree was reconstructed using FastME v2.1.4 [59] based on the matrix, with *F. hirta* designated as the outgroup.

To reconstruct the chloroplast phylogeny, we first constructed chloroplast consensus for 18 individuals. Whole-genome resequencing data from 18 individuals and publicly available whole-genome resequencing data for *F. hirta* (SAMC1044464) were mapped to the *F. hispida* chloroplast genome (https://download.cncb.ac.cn/gwh/Plants/Ficus_hispida_plant_cpDNA_seq_260_GWHABVF01000000) using BWA v0.7.17 [51]. We then performed Variant Calling using SAMTOOLS v.1.16 [52]. We used coverage to distinguish plastid and nuclear sequences. For each position in the reference chloroplast genome, bases were called if the coverage depth exceeded 300 and if more than 90% of the reads supported either the reference or an alternate base. Any position not meeting these criteria was treated as missing data, and InDels were excluded from all analyses. We reconstructed a maximum likelihood (ML) phylogeny using IQ-TREE v.2.1.2 [60] from a concatenated alignment of the complete chloroplast genomes from 19 samples, with a total length of 160,276 bp.

### Morphological analyses

The fruiting phenology of *F. hispida*, *F. squamosa*, and putative hybrids was monitored in the sympatric study area during the rainy season. Trees were checked at intervals of 1–2 weeks over a 10-week period. For each observed tree, we recorded the presence or absence of figs in key developmental phases, specifically the receptive phase (B phase) in both male and female trees, and the wasp emergence phase (D phase) of male trees. Morphological measurements were conducted on 14 individuals of *Ficus hispida*, 25 *Ficus squamosa*, and four of the putative hybrids. The morphological dataset included a larger number of individuals than the genomic dataset to enhance statistical power. It comprised all 12 genotyped individuals of the parent species (6 of each *F. hispida* and *F. squamosa*) plus 8 additional individuals of *F. hispida* and 19 additional individuals of *F. squamosa*. For the putative hybrids, the morphological analysis included four of the six genotyped individuals (two genotyped hybrids were saplings and excluded from morphological measurements).

Leaf measurements were performed on mature leaves at the Queen Sirikit Botanic Garden (QSBG). For each individual, seven mature and healthy leaves were selected for analysis. Measured traits included leaf length (excluding petiole), maximum leaf width (at the widest point), and petiole length. Leaf area was calculated as the product of leaf length and maximum leaf width, adjusted by a correction factor (CF) for their ovate leaf shape [61]. The leaf ratio was determined by dividing leaf length by leaf width, while vein count was recorded as the number of lateral veins on one side of the midrib. After measurement, leaf samples were oven-dried at 70 °C for 72 h to determine leaf dry mass, and specific leaf area (SLA) was calculated by dividing leaf area by leaf dry mass. Stomata were observed using the tape-peel method [62] based on three random, nonoverlapping views from seven healthy leaves for each individual. The stomatal density in 1 mm2 was observed under the microscope (Nikon Digital Sight DF-Fi3). Thirty stomata were randomly selected using a microscope at 400× magnification with the integrated digital camera system (Nikon Digital Sight DF-Fi3). Then stomatal width and length were measured at 400× magnification to calculate stomatal size. Images were analyzed using ImageJ version 1.54 g [63].

The normality of data for each morphological trait was assessed using the Shapiro-Wilk test. As the data for most traits significantly deviated from a normal distribution, non-parametric tests were employed. Statistical differences among the three taxa for each trait were assessed using the Kruskal-Wallis test. For traits showing a significant difference, pairwise post-hoc comparisons were conducted using Wilcoxon rank-sum tests. Data were analyzed and visualized in R-4.4.1 [64]. Box plots visualizing the variation in each morphological trait among the three taxa (as shown in Fig. 6) were generated using the *‘ggplot2’* package (version 3.5.1). Multidimensional scaling (MDS) was performed on the morphological measurements using the ‘*cmdscale’* function. To further evaluate the discriminatory power of the leaf traits and classify individuals among the three groups, we conducted a discriminant function analysis (DFA) using the ‘*lda’* function.

## Results

### Genomic analyses

**Fig. 3:**
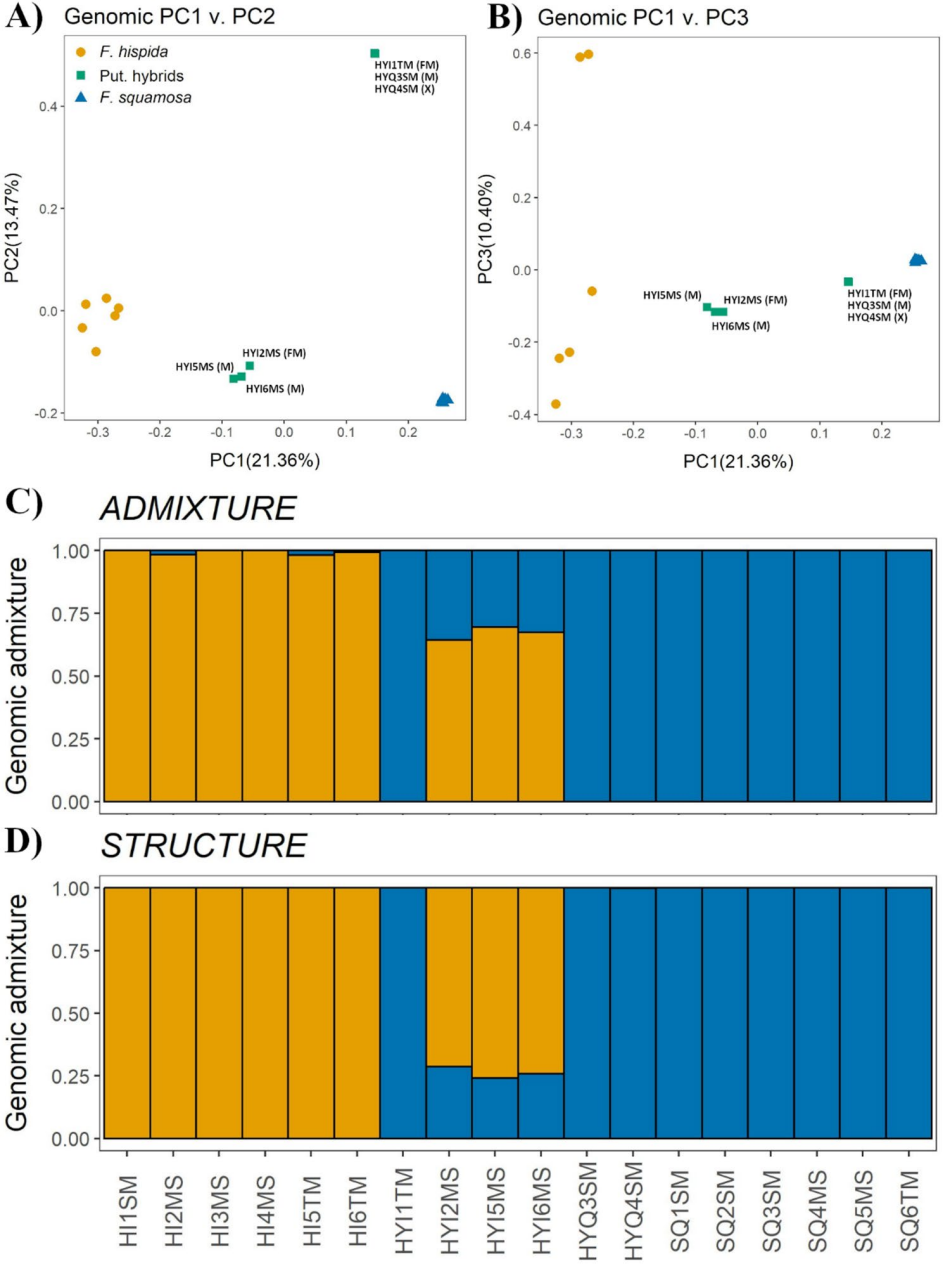
Principal component analysis of 12,046 SNPs, (**A**) PC1 v. PC2 explained 34.83%; PC1: 21.36% and PC2: 13.47% and (**B**) PC1 v. PC3 explained 31.76%; PC1: 21.36% and PC3: 10.40%. Population genomic admixture of three taxa from (**C**) ADMIXTURE and (**D**) STRUCTURE software, samples at *K* = 2 (from left to right: *F. hispida*, putative hybrids and *F. squamosa*)

Principal component analysis of SNP data clearly separated the parent species along PC1, which explained 21.36% of the genetic variance. The putative hybrid individuals formed a distinct cluster intermediate between the *F. hispida* and *F. squamosa* clusters along this axis (Fig. 3A, B). Both ADMIXTURE and STRUCTURE analyses at K = 2 confirmed the hybrid status of the three intermediate individuals (HYI2MS, HYI5MS, HYI6MS), showing they are admixed with a greater proportion of genetic material from *F. hispida* (Fig. 3C, D). The ancestry proportions in these hybrids (approximately 20–30% *F. squamosa* ancestry) are consistent with them being later-generation hybrids (e.g., F2 or backcrosses) rather than F1 hybrids, which would be expected to have ~ 50% ancestry from each parent. The other three putative hybrids (HYI1TM, HYQ3SM, HYQ4SM) showed ancestry almost entirely assigned to *F. squamosa*, suggesting they may be late-generation backcrosses. In addition, in the field, these three putative hybrids exhibit morphologically intermediate traits but visually are more similar to *F. squamosa* (RK personal observation).

**Fig. 4:**
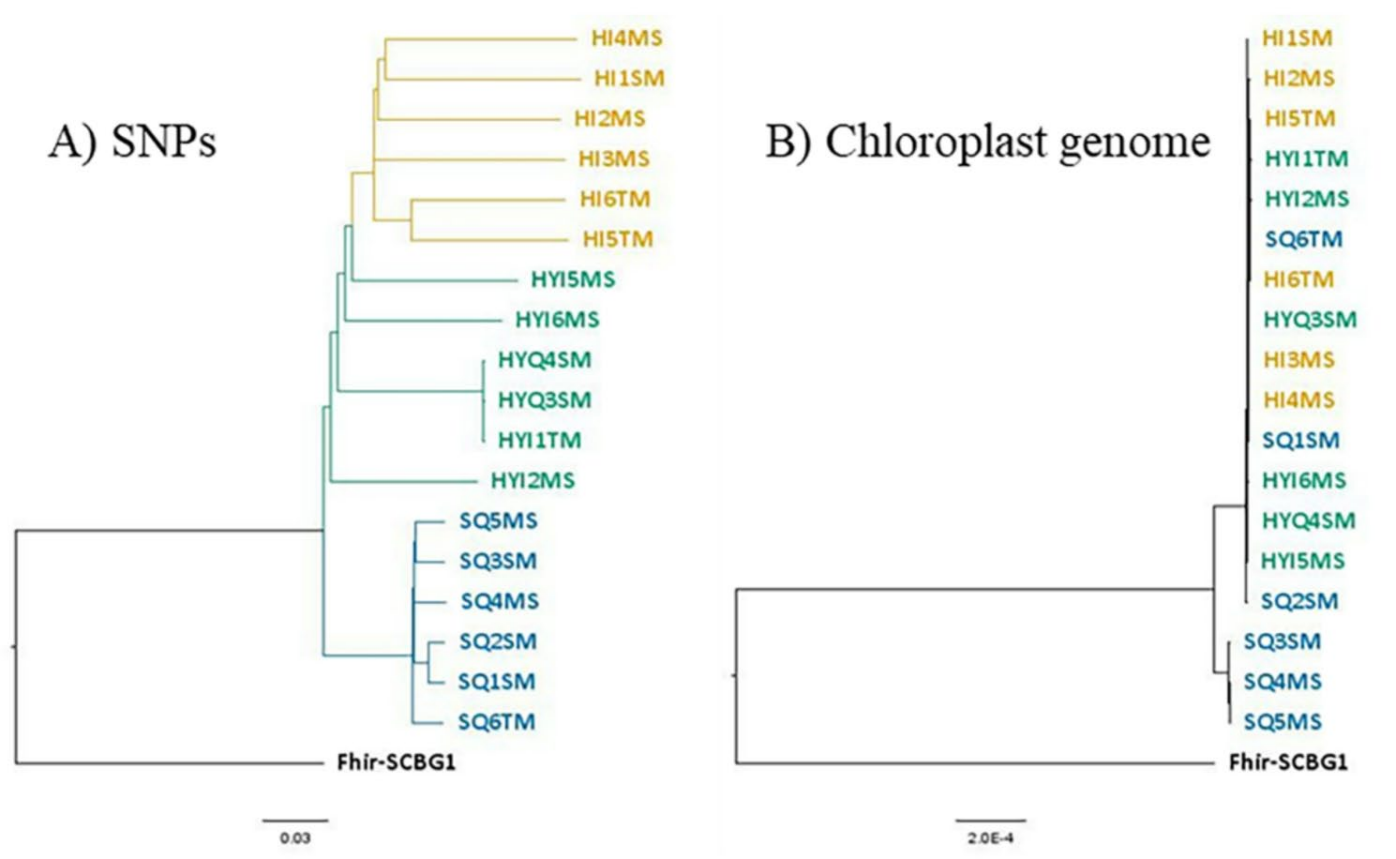
Phylogenetic tree constructed by (**A**) neighbour joining (NJ) method using 12,046 SNPs and (**B**) maximum likelihood (ML) method using chloroplast genome

Phylogenetic analysis of nuclear SNPs also separated the parent species and grouped the putative hybrids together on a distinct branch (Fig. 4A). To investigate the history of hybridization, we constructed a phylogeny from chloroplast genomes. This analysis grouped all hybrids and some *F. squamosa* individuals within the *F. hispida* clade (Fig. 4B), suggesting that *F. hispida* was the maternal parent in the original hybridization events and indicating historical or ongoing asymmetric introgression.

### Fruiting phenologies

Field observations during Thailand’s rainy season (June to September) revealed periods of temporal overlap in the fruiting phases of *F. hispida*, *F. squamosa*, and their hybrids in sympatric populations. As shown in Figure S1, the receptive phase (B-phase) and wasp emergence phase (D-phase) of male trees overlapped at various times, though not continuously, during the 14-week observation period. These overlapping phases were documented between the hybrid individuals and both parent species. Both parent species showed increased fig production during the rainy season, and male trees of both species maintained asynchronous fig production, providing potential opportunities for pollinator movement throughout the cycle.

### Morphological analyses

Morphological analyses revealed a range of traits that were intermediate in the hybrids compared to the parent species, *F. hispida* and *F. squamosa*. A summary of key diagnostic characteristics for growth form, leaf texture, and syconium morphology is provided in Table 1, with representative individuals shown in Fig. 2 and Figure S3.

**Table 1:** Characteristics of leaves and figs of the three groups

Species	Habits	Leaf texture	Fig/Syconium	Fig position
*F. hispida*	Tree (up to 13 m tall)	Papery covered with a lot of white hair	Globose. Both male and female figs change from bright-green to yellow when ripe.	Cauliflorous and ramiflorous
*F. squamosa*	Shrub, stolon-like root (Rheophytes)	Leathery, covered with slightly golden-brown hair along the midrib and veins	Rhombic-ovoid. Both male and female figs change from dark-green to yellow when ripe.	Cauliflorous and rooting stolons near or under the soil
Putative hybrids	Shrub, small tree	Papery covered with slightly white hair and golden-brown hair present on the midrib	Rhombic-ovoid. Both male and female figs change from green to yellow when ripe.	Cauliflorous, ramiflorous, and rooting stolons near or under the soil

**Fig. 5:**
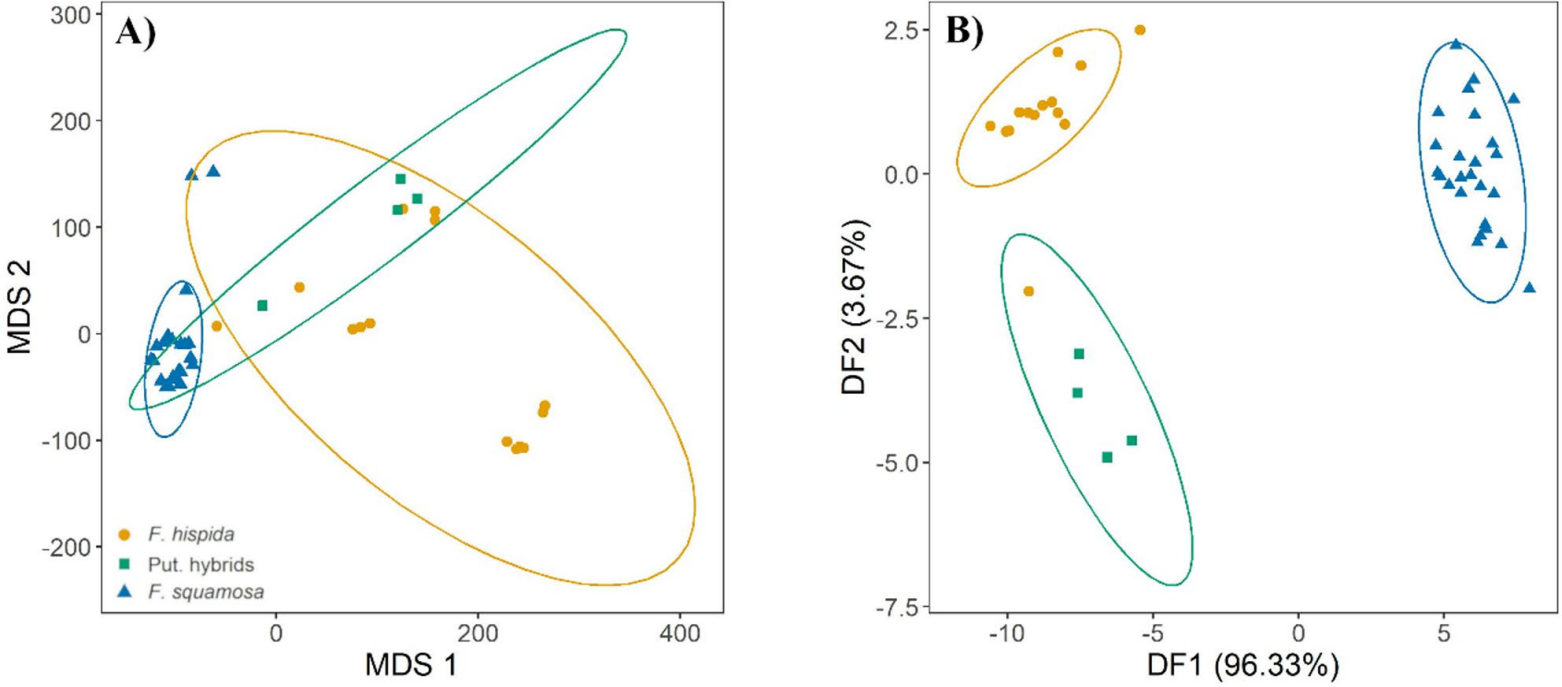
(**A**) Multidimensional scaling (MDS) of leaf morphology data, elevation, and distance from stream of the putative hybrids and parents (stress value = 0.16). (**B**) discriminant function analysis (DFA). *F. hispida* are shown in yellow circles, putative hybrids are shown in green triangles and *F. squamosa* are shown in blue squares. Ellipses represent a confidence interval (95%)

Multidimensional scaling of the leaf morphometric dataset, elevation, and distance from streams showed an overlapping area with hybrids as intermediate between *F. hispida* and *F. squamosa* displaying a combination of traits from *F. hispida* and *F. squamosa* (Fig. 5A). Discriminant function analysis (DFA) showed that leaf size including leaf length, leaf width, leaf ratio, and leaf area have a strong negative and positive loading on both discriminant functions (DF1 and DF2) where DF1 explained 96.33% of the variance and DF2 explained 3.67% of the variance (Table 2). This indicated that leaf size or shape of the three taxa are different but putative hybrids and *F. hispida* were loaded on negative side which indicated that their hybrid leaf traits were closer to *F. hispida* (Fig. 5B), but they are nonetheless difficult to identify when growing in natural habitats.

We also examined the relationship between proximity to streams and the morphological characteristics of the leaves (results are shown in supporting data, Figures S4 and S5). Spearman’s rank correlation coefficient was used to evaluate the strength and direction of the relationship between distance from the stream and each leaf trait. This analysis helps elucidate how leaf characteristics may vary along environmental gradients, such as moisture availability, which is often influenced by proximity to a water source. Certain traits, such as leaf ratio, showed a strong negative correlation, indicating that plants with smaller and narrower leaves were more likely to grow near the stream. Similarly, stomatal density exhibited a strong negative correlation with distance from the stream, suggesting that plants growing closer to the stream had a higher stomatal density (Figure S4).

**Table 2:** Discriminant function analysis (DFA) of leaf traits. Eigenvalues, percent variance, and the standardized discriminant function coefficients of functions

	Functions	
	DF1	DF2
Eigenvalue	1083.21	41.26
Percent variance (%)	96.33	3.67
Elevation	-1.38	-0.03
Distance from stream	-1.95	-1.13
Leaf length	-4.16	6.61
Leaf width	-6.79	14.90
Leaf ratio	5.99	-0.51
Vein count	-0.38	-2.30
Petiole length	-2.97	-0.41
Leaf area	5.67	-9.70
Specific leaf area	-2.37	-2.08
Stomatal size	0.79	-8.04
Stomatal density	0.41	-4.00

**Fig. 6:**
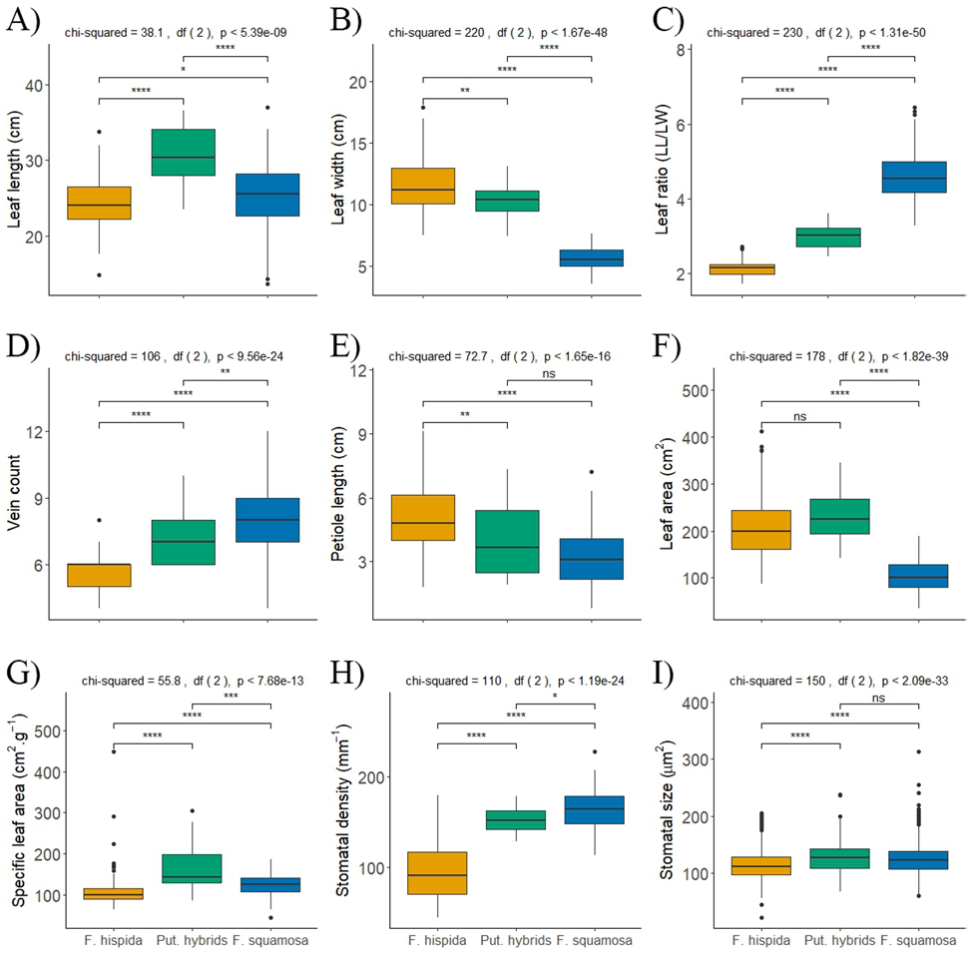
Box plots of leaf morphology showing (**A**-**E**) leaf length, leaf width, leaf ratio, vein count, and petiole length; (**F**-**I**) leaf area, specific leaf area, stomatal density, and stomatal size, respectively. Statistical significance of differences among taxa was assessed using the Kruskal-Wallis test, followed by pairwise Wilcoxon tests (‘*’ *p* < 0.05; ‘**’ *p* < 0.01; ‘***’ *p* < 0.001; ‘****’ *p* < 0.0001; ‘ns’ *p* ≥ 0.05)

The box plots for leaf and stomata traits suggested that putative hybrids have intermediate leaf morphology and features that may be helpful in identifying putative hybrids (Fig. 6). Leaf width, leaf ratio, vein count, and petiole length of the hybrids was intermediate between *F. hispida* and *F. squamosa* (Fig. 6B, C, D and E), but leaf length, leaf area and specific leaf area of the hybrids appears to be larger than that of both *F. hispida* and *F. squamosa* (Fig. 6A, F and G). hybrids may tend to grow-faster than both parents, whereas stomatal area and stomatal density of the hybrids is closer to that of *F. squamosa* (Fig. 6H and I). Measurements from a larger sample group may help identify practical traits to distinguish hybrids from the parents.

## Discussion

The complex interplay between fig species and their pollinators provides insights into plant speciation mechanisms in general, but particularly among plants that have intimate associations with their pollinators. Our analysis of hybridization between *Ficus hispida* and *F. squamosa* confirms that reproductive barriers between closely related *Ficus* species can be bridged, most likely via pollinator host-switching, and that significant ecological differences are no barrier to the production of hybrids.

### Evidence for natural hybridization, hybrid characteristics and gene flow patterns

Field observations revealed phenological overlap between sympatric populations of *F. hispida* and *F. squamosa* and their hybrids in, particularly during Thailand’s rainy season (June to September). These species exhibited concurrent presence of receptive (B phase) and mature (D phase) figs on male and female trees (Figure S1), with increased fig production on adult female trees during this period.

These observations align with previous studies demonstrating temporal overlap in the reproductive phenology of the species. Male trees of both *F. hispida* and *F. squamosa* show peak fig production during the hot/dry season but female trees peak in the rainy season, with *F. hispida* exhibiting synchronized peaks in wasp emergence and seed production during May, coinciding with *F. squamosa*’s primary fig initiation period. Male trees of both species maintain asynchronous fig production, though with varying degrees of continuity throughout the annual cycle [44, 65].

The temporal concordance in reproductive events, particularly the overlap of receptive and mature figs (phase B and D of male fig), creates opportunities for interspecific pollinator transfer. The extended periods of fig availability on male trees of both species provide continuous resources for fig wasps, potentially facilitating pollinator host shifts in sympatric populations.

The contrasting phenological patterns appear to reflect species-specific adaptations for maintaining pollinator populations, with *F. hispida* showing coordinated male and female fig production peaks, while *F. squamosa* exhibits more pronounced sexual differentiation in reproductive timing. Yet, the presence of hybrids indicates that these phenological strategies, while potentially reinforcing reproductive isolation, do not completely prevent gene flow between species when their distributions overlap.

The combination of temporal overlap in fig development phases and joint increased fig production during the rainy season may create conditions conducive to hybridization events in sympatric populations of *F. hispida* and *F. squamosa*, despite the presence of apparent ecological barriers to gene flow. While both species exhibit peak reproductive activity during similar seasonal windows, they show distinct habitat preferences, with *F. squamosa* requiring riparian environments and *F. hispida* more of a generalist. This spatial segregation might typically limit opportunities for interspecific pollinator movement, depending on the mobility of the pollinators. However, the discovery of hybrid individuals suggests that habitat segregation alone has been insufficient to maintain complete reproductive isolation.

Hybrid individuals between *F. hispida* and *F. squamosa* display intermediate traits between these parent species, particularly in leaf characteristics and fig morphology (Figures S2 and S3). This morphological intermediacy, a classical but not inevitable signature of hybridizationaligns with similar patterns observed in other *Ficus* species, such as *F. septica* and *F. fistulosa* on the Krakatau Islands [32] and *F. aculeata* and *F. coronulata* in Australia [34].

Our genomic evidence, particularly from genomic admixture and phylogenetic trees, revealed a patterns of genetic exchange where some hybrids had a greater genetic contribution from *F. hispida*. This suggests multiple generations of backcrossing. This pattern of repeated hybridization and introgression again aligns with observations in some other fig species [34, 36, 66].

### Pollinator host switching and environmental pressure

Machado et al. [13] and Moe et al. [67] provided early evidence challenging strict one-to-one specificity, while recent genomic studies by Wang et al. [31] such that hybridization may have been prevalent throughout the evolutionary history of this mutualism. Our results are consistent with a recent study by Satler et al. [66] of the genetic consequences of pollinator and host sharing in Panamanian Monoeciousfigs and their associated pollinator wasps. They found that hybridization and introgression occurs among their fig species, likely due to shared pollinators and host plants. However, these processes were not observed in the pollinator wasps, suggesting that the wasps maintain stronger reproductive isolation despite sharing hosts. The genetic structure and potential hybridization within the *Ficus auriculata* species group was investigated using genetic markers [16] They found significant genetic differentiation among the species, indicating that they retain distinct genetic identities despite sharing pollinator wasps. Although some evidence of hybridization was detected, it was relatively rare, suggesting strong reproductive barriers exist between these species.

The breakdown of pollinator specificity between *F. squamosa* and *F. hispida* may be driven by multiple interacting factors. Environmental pressures, particularly flash floods affecting *F. squamosa* populations [45], create scenarios where pollinator wasps must adapt their host choices. This situation is exacerbated by the short lifespan of pollinating wasps ranges from a few hours to a few days [68], indicating the necessity for figs to be available for pollination within this narrow timeframe [15] and the phenological patterns between species, with *F. hispida* populations producing figs continuously [65, 69]. provides opportunities for pollinator-mediated interactions between the two fig species [69]. In addition, recent analyses of volatile organic compounds (VOCs) in these species reveal notable chemical similarities alongside significant interspecific differences [37, 40]. Shared attractant volatiles facilitating cross-pollination have been documented in other fig species pairs, including *F. natalensis* and *F. burkei* [70], *F. microdictya* and *F. umbrae* [71], *F. hirta* and *F. triloba* [18], and within the *F. auriculata* complex [25].

### Evolutionary and conservation implications

The role of hybridization in plant evolution has been increasingly recognized, from its contribution to adaptive radiation [5] to its importance in responding to environmental gradients [20]. Our findings contribute to the broader understanding of hybridization as “windows on evolutionary process” [3], A perspective that is particularly relevant in systems with specialized pollinators [40], .where pollinator-mediated hybridization can facilitate genetic exchange even in highly specialized mutualisms.

Several key questions warrant further investigation in our study system: (1) the long-term fitness consequences of hybridization and backcrossing, particularly in light of patterns observed in other fig species [72, 73]; (2) the specific chemical compounds influencing pollinator host choice, building on recent VOCs analyses [37]; and (3) the role of hybrids that are apparently restricted to intermediate soil conditions an dits significance in preserving genetic diversity under climate change [39], . Future research should aim to expand sample sizes to better characterize their distributions to confirm they are restricted to certain environmental conditions to assess the fitness implications of backcrossing and explore pollinator behavior and host choice dynamics.

Understanding these aspects will be necessary for understanding the evolutionary trajectory of fig-pollinator systems under changing environmental conditions. As demonstrated by recent studies of closely related fig species [27, 39, 40], such research is particularly relevant in biodiversity hotspots such as South East Asia’s forests, where environmental changes appear likely to alter long-established species interactions. Such changes may result directly from climate change, but also indirectly via their impact on human populations. For example, habitat generalists like *Ficus hispida* seem likely to benefit from the anthropogenic loss of riparian forest cover, bringing them more frequently into close contact with populations of *F. squamosa.*

## Conclusions

This study provides compelling evidence for hybridization having taken place between *Ficus hispida* and *F. squamosa*. The presence of hybrids, combined with the known pollinator sharing within this species complex, strongly suggests that pollinator host-switching is the most likely mechanism bridging reproductive barriers between these closely related fig species. Through comprehensive morphological and genomic analyses, we have documented patterns of intermediate traits in hybrid individuals, with genomic analyses revealing complex patterns of backcrossing, particularly with *F. hispida*. The detection of both recent hybrids and introgression through chloroplast genome indicates that hybridization has been a relatively recent phenomenon. Such hybridization may have a role in facilitating future adaptation to changing environments, a role that is particularly relevant in the context of ongoing climate change and habitat disturbance.

## Supplementary Information

Below is the link to the electronic supplementary material.

Supplementary Material 1

## Data Availability

The datasets supporting this study are available from the corresponding author upon reasonable request. Raw sequencing data generated for genome resequencing have been deposited in the National Center for Biotechnology Information (NCBI) under BioProject accession number PRJNA1244258. Genomic sequencing data is available at https://www.ncbi.nlm.nih.gov/sra/PRJNA1244258.
