# Supplementary material for: Hybridization and introgression between two fig trees with contrasting ecological preferences
Source: BMC Ecol Evol. 2025 Dec 24;25:141. doi: 10.1186/s12862-025-02476-7 (PMC12729013; doi:10.1186/s12862-025-02476-7)
Supplement: Supplementary file 1 — Supplementary Material 1 [file 12862_2025_2476_MOESM1_ESM.docx]

**Supplementary Information**

**Hybridization and introgression between two fig trees with contrasting ecological preferences**

**Ramil Kohkaew^1,2^, Yamei Ding^1^, Pornwiwan Pothasin^3^, Nattaya Srisawad^4^, Stephen G. Compton^5^, and Hui Yu^1,6*^**

^1^State Key Laboratory of Plant Diversity and Specialty Crops, South China Botanical Garden, Chinese Academy of Sciences, Guangzhou, Guangdong 510650, China

^2^University of Chinese Academy of Sciences, Beijing 100049, China

^3^Conservation Biology Program, School of Interdisciplinary, Mahidol University, Kanchanaburi Campus, Kanchanaburi 71150, Thailand

^4^Insititute of Molecular Biosciences, Mahidol University, Nakhon Pathom, 73170, Thailand

^5^School of Biology, University of Leeds, Leeds, LS2 9JT, United Kingdom

^6^Guangdong Provincial Key Laboratory of Applied Botany, South China Botanical Garden, Chinese Academy of Sciences, Guangzhou, Guangdong 510650, China

***Correspondence:** Hui Yu: [yuhui@scbg.ac.cn](mailto:yuhui@scbg.ac.cn)

**Table S1**. Tree height, basal area (means ± SD), and elevation of the three taxa. Basal area refers to the cross-sectional area of a tree's stem at root collar (5 cm above ground). It indicates tree size, and competition among species, with higher values reflecting larger trees and greater resource competition. Basal area is also used to estimate forest biomass, productivity, and carbon storage.

| **Species** | **Tree height**  **(m)** | **Basal area**  **(m^2^)** |  | **Elevation (m)** |  |
| --- | --- | --- | --- | --- | --- |
|  |  |  | **Min** | **Max** | **Median** |
| *F. hispida* | 6.40 ± 1.62  (n=24) | 1.923 ± 1.278  (n=24) | 333.0 | 940.0 | 492.0 |
| Put. hybrids | 3.30 ± 1.48  (n=6) | 0.024 ± 0.011  (n=6) | 351.0 | 627.0 | 487.5 |
| *F. squamosa* | 0.69 ± 0.28  (n=18) | 0.007 ± 0.005  (n=18) | 346.0 | 561.0 | 395.5 |

**Table S2**. Distance from individuals to stream and distance from putative hybrids to parents.

| **Species** | **Distance from stream (m)** | | | **Distance from parents (m)** | | | | | |
| --- | --- | --- | --- | --- | --- | --- | --- | --- | --- |
|  |  |  |  | ***F. hispida*** | | | ***F. squamosa*** | | |
|  | **Min** | **Max** | **Median** | **Min** | **Max** | **Median** | **Min** | **Max** | **Median** |
| *F. hispida*  (n=32) | 25.4 | 215.9 | 218.9 | - | - | - | - | - | - |
| Put. hybrids  (n=6) | 0 | 300.1 | 101.1 | 4.6 | 334.5 | 85.5 | 2.0 | 1,216.0 | 314.7 |
| *F. squamosa*  (n=34) | 0 | 0 | 0 | - | - | - | - | - | - |

**Table S3**. Location of 18 individuals that leaves samples were collected for genome sequencing in this study.

| **Code** | **Species** | **Sex** | **Location** | **Latitude** | **Longitude** | **Note** |
| --- | --- | --- | --- | --- | --- | --- |
| HI1SM | *F.hispida* | Female | Sai Mok WF | 19° 0' 49.451" N | 98° 50' 23.645" E |  |
| HI2MS | *F.hispida* | Female | Mae Sa WF | 18° 54' 28.718" N | 98° 53' 23.243" E |  |
| HI3MS | *F.hispida* | Male | Mae Sa WF | 18° 54' 28.417" N | 98° 54' 5.735" E |  |
| HI4MS | *F.hispida* | Male | Mae Sa WF | 18° 54' 27.279" N | 98° 54' 8.232" E |  |
| HI5TM | *F.hispida* | Female | Tat Mok WF | 18° 57' 53.749" N | 98° 55' 9.461" E |  |
| HI6TM | *F.hispida* | Female | Tat Mok WF | 18° 57' 53.716" N | 98° 55' 9.803" E |  |
| HYI1TM | Putative hybrids | Female | Tat Mok WF | 18° 56' 48.372" N | 98° 52' 25.752" E |  |
| HYI2MS | Putative hybrids | Female | Mae Sa WF | 18° 53' 59.748" N | 98° 53' 0.96" E |  |
| HYQ3SM | Putative hybrids | Male | Sai Mok WF | 18° 53' 59.748" N | 98° 53' 0.974" E | Sapling |
| HYQ4SM | Putative hybrids | No data | Sai Mok WF | 19° 0' 59.491" N | 98° 50' 47.136" E | Sapling |
| HYI5MS | Putative hybrids | Male | Mae Sa WF | 18° 54' 27.468" N | 98° 53' 21.984" E |  |
| HYI6MS | Putative hybrids | Male | Mae Sa WF | 18° 54' 27.252" N | 98° 53' 22.092" E |  |
| SQ1SM | *F.squamosa* | Female | Sai Mok WF | 19° 0' 59.165" N | 98° 50' 46.247" E |  |
| SQ2SM | *F.squamosa* | Female | Sai Mok WF | 19° 1' 0.11" N | 98° 50' 47.546" E |  |
| SQ3SM | *F.squamosa* | Male | Sai Mok WF | 19° 1' 0.24" N | 98° 50' 47.751" E |  |
| SQ4MS | *F.squamosa* | Female | Mae Sa WF | 18° 54' 30.801" N | 98° 54' 23.51" E |  |
| SQ5MS | *F.squamosa* | Female | Mae Sa WF | 18° 54' 30.703" N | 98° 54' 23.442" E |  |
| SQ6TM | *F.squamosa* | No data | Tat Mok WF | 18° 57' 34.798" N | 98° 51' 12.731" E |  |

**Table S4**. Means (± SD) of leaf trait of the three taxa.

| **Species** | ***n*** | **Leaf length (cm)** | **Leaf width (cm)** | **Leaf ratio (LL/LW)** | **Vein count** | **Petiole length (cm)** | **Leaf area (cm^2^)** | **Specific leaf area (Cm^2^.g^-1^)** |
| --- | --- | --- | --- | --- | --- | --- | --- | --- |
| *F. hispida* | 14 | 24.56 | 11.64 | 2.14 | 5.83 | 5.09 | 211.10 | 110.41 |
|  |  | (± 3.63) | (± 2.35) | (± 0.22) | (± 1.03) | (± 1.64) | (± 72.47) | (± 47.38) |
| Put. hybrids | 4 | 30.75 | 10.33 | 2.99 | 7.11 | 4.02 | 231.18 | 163.86 |
|  |  | (± 3.88) | (± 1.35) | (± 0.31) | (± 1.13) | (± 1.70) | (± 55.36) | (± 54.38) |
| *F. squamosa* | 25 | 26.07 | 5.63 | 4.66 | 8.17 | 3.31 | 107.67 | 126.25 |
|  |  | (± 4.37) | (± 0.91) | (± 0.62) | (± 1.73) | (± 1.24) | (± 31.94) | (± 26.51) |

**Table S5**. Means (± SD) of stomatal trait of the three taxa.

| **Species** | ***n*** | **Stomatal width (µm)** | **Stomatal length (µm)** | **Stomatal area (µm^2^)** | **Stomatal density (mm^-1^)** |
| --- | --- | --- | --- | --- | --- |
| *F. hispida* | 14 | 10.43 | 13.76 | 113.54 | 96.56 |
|  |  | (± 1.36) | (± 1.86) | (± 25.62) | (± 33.01) |
| Put. hybrids | 4 | 11.22 | 14.47 | 128.09 | 153.05 |
|  |  | (± 1.30) | (± 2.08) | (± 26.97) | (± 14.61) |
| *F. squamosa* | 25 | 11.35 | 13.93 | 124.91 | 163.15 |
|  |  | (± 1.32) | (± 1.67) | (± 25.24) | (± 23.92) |


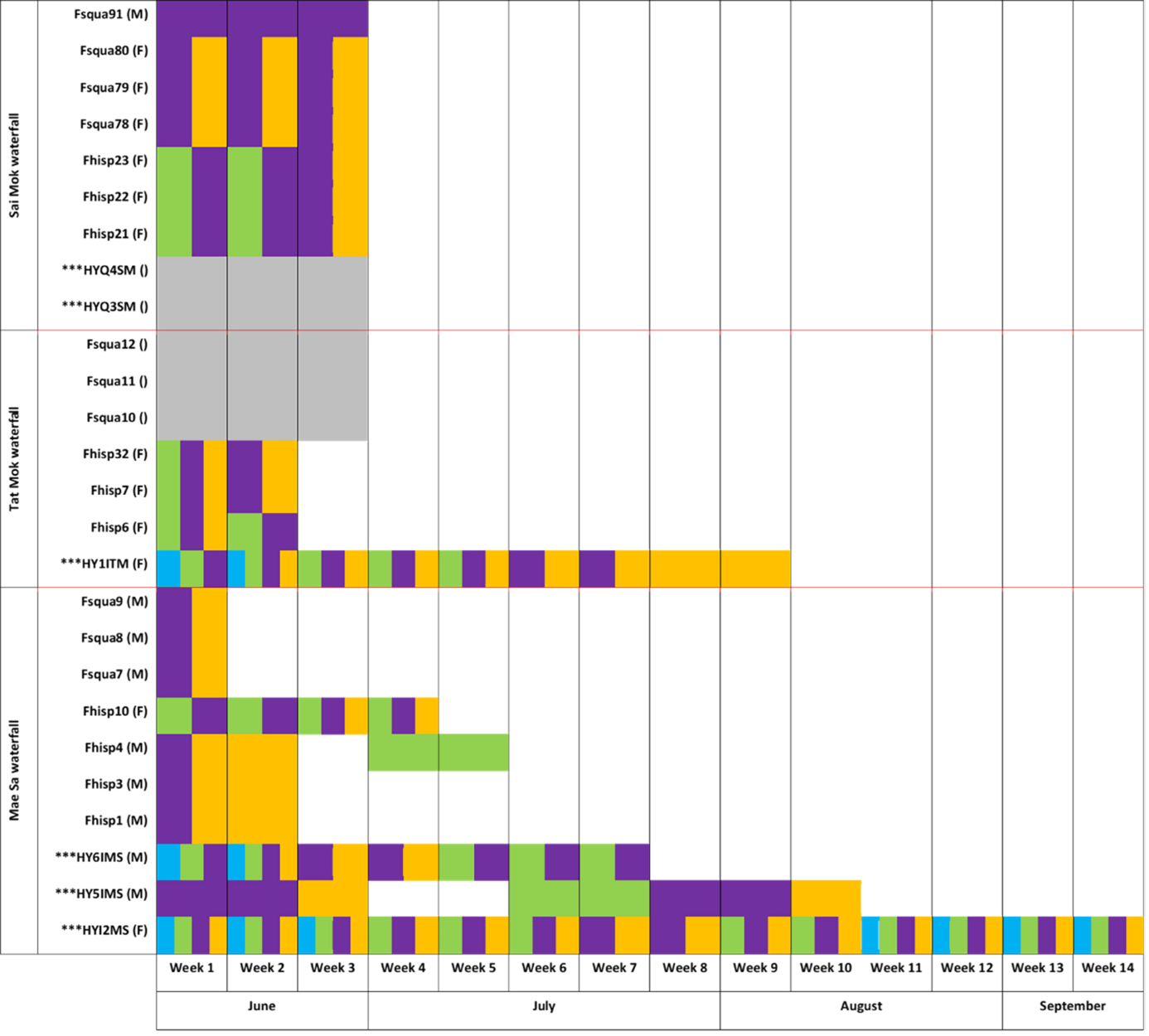


**Figure S1**. Occurrence of difference phase of fig in Chiang Mai, Thailand for all study sites that found *Ficus* *squamosa* (Fsqua), *F*. *hispida* (Fhisp), and putative hybrid (HYI and HYQ) in sympatric area ((F): female, (M): male, ( ): no data). Each row represents different sampling tree and redline separate sampling site: Sai Mok waterfall, Tat Mok waterfall, and Mae Sa waterfall. A total of 10 *F. squamosa* trees, 10 *F. hispida* trees, and 6 putative hybrid trees. The colour bar represents fig development stages: phase A (blue), phase B (green), phase C (purple), and phase D and E (orange). The grey bar represents no fig found. Throughout observation there are overlapping occurrence between B and D phase of fig among three taxa. (‘***’ putative hybrids that were collected leaf samples for genome sequencing)


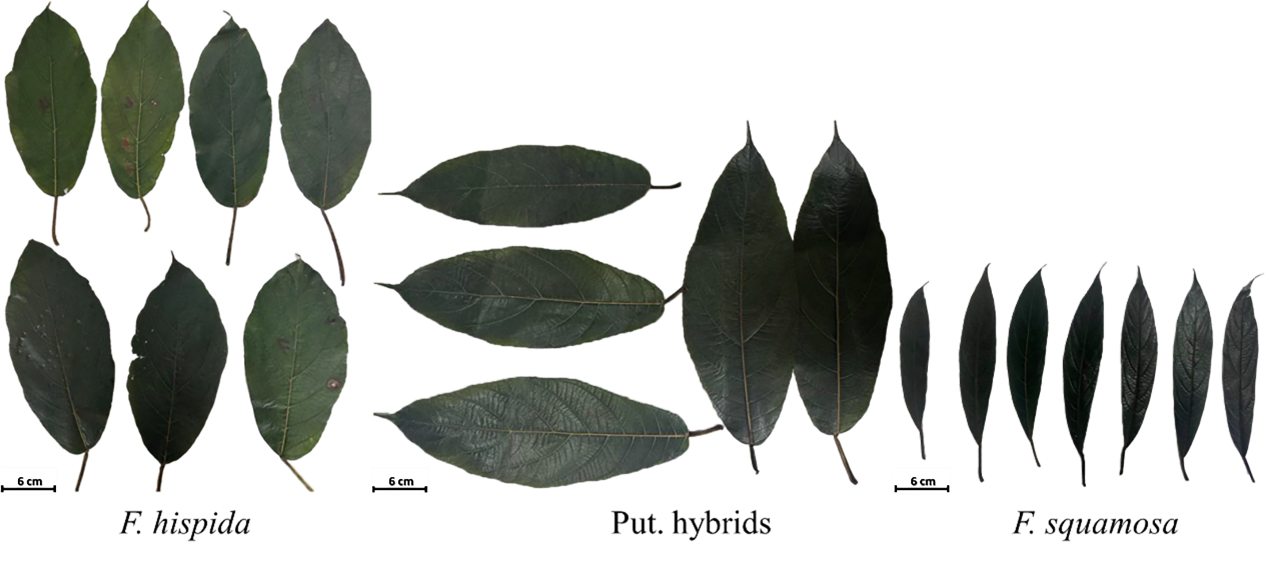


**Figure S2**. Example leaves of the three taxa. Bar = 6 cm.


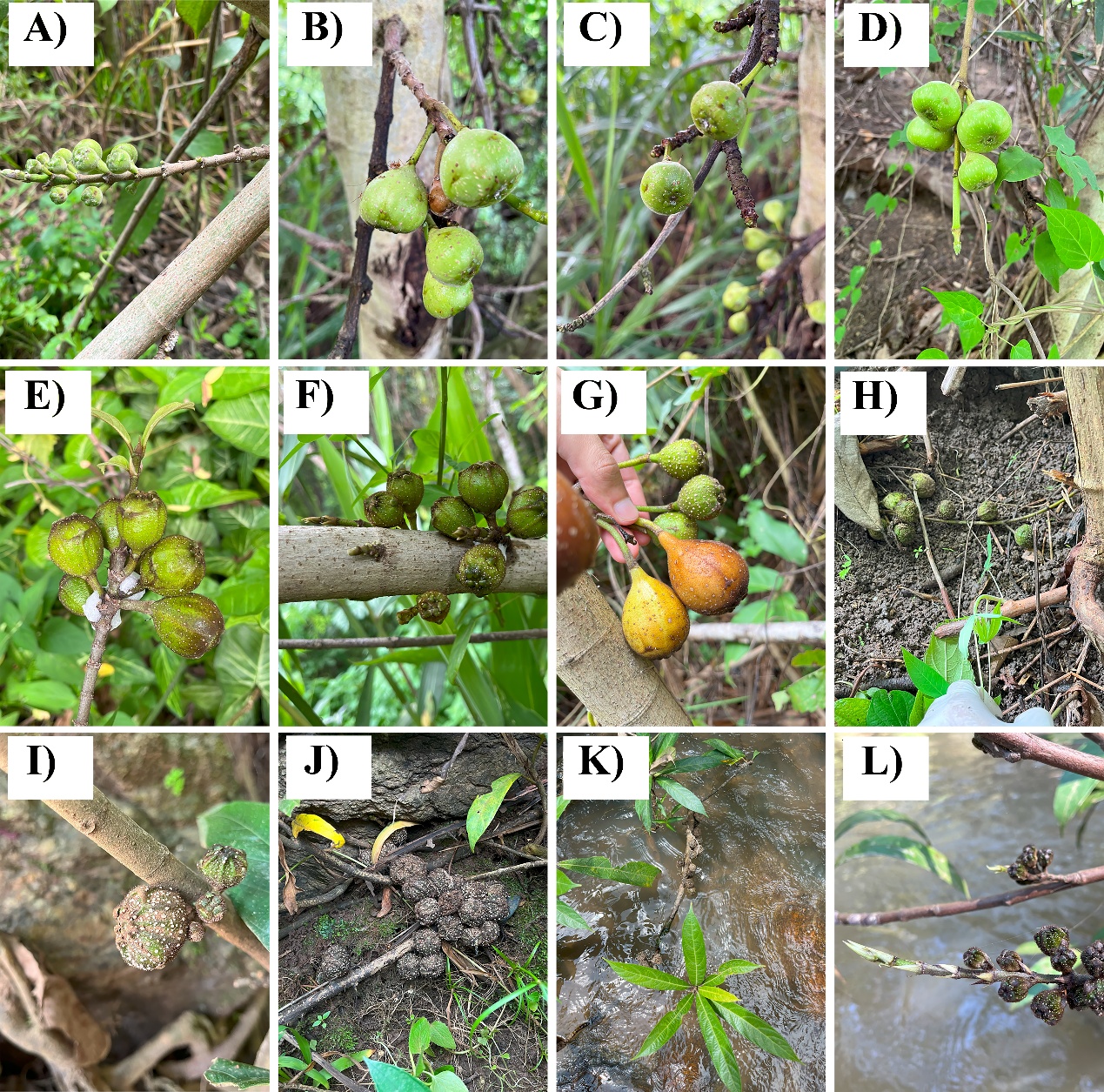


**Figure S3**. Syconium of the three taxa. (A-D) adult individuals, cauliflorous and ramiforous figs along the trunk of *F. hispida*, (E-H) adult individuals, cauliflorous, ramiforous, and rooting stolon near or under the ground figs of putative hybrids, and (I-L) adult individuals, cauliflorous and rooting stolon near or under the ground figs of *F. squamosa*.

**
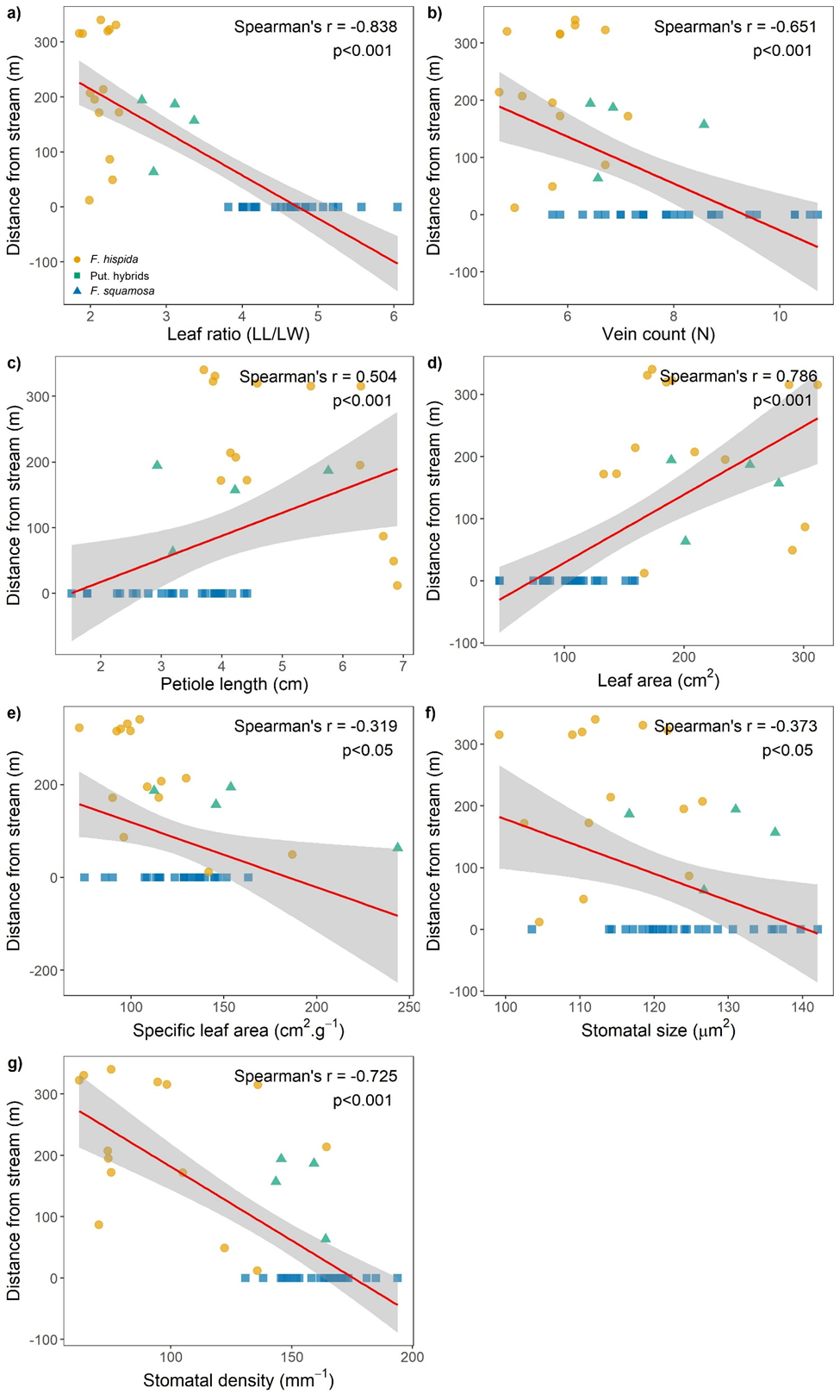
**

**Figure S4**. Spearman’s rank correlation between distance from the stream and other relevant leaf traits: (a) leaf ratio, (b) vein count, (c) petiole length, (d) leaf area, (e) specific leaf area, (f) stomatal size, and (g) stomatal density. *F. hispida* are shown in yellow circle, putative hybrids are shown in green triangle and *F. squamosa* are shown in blue square.


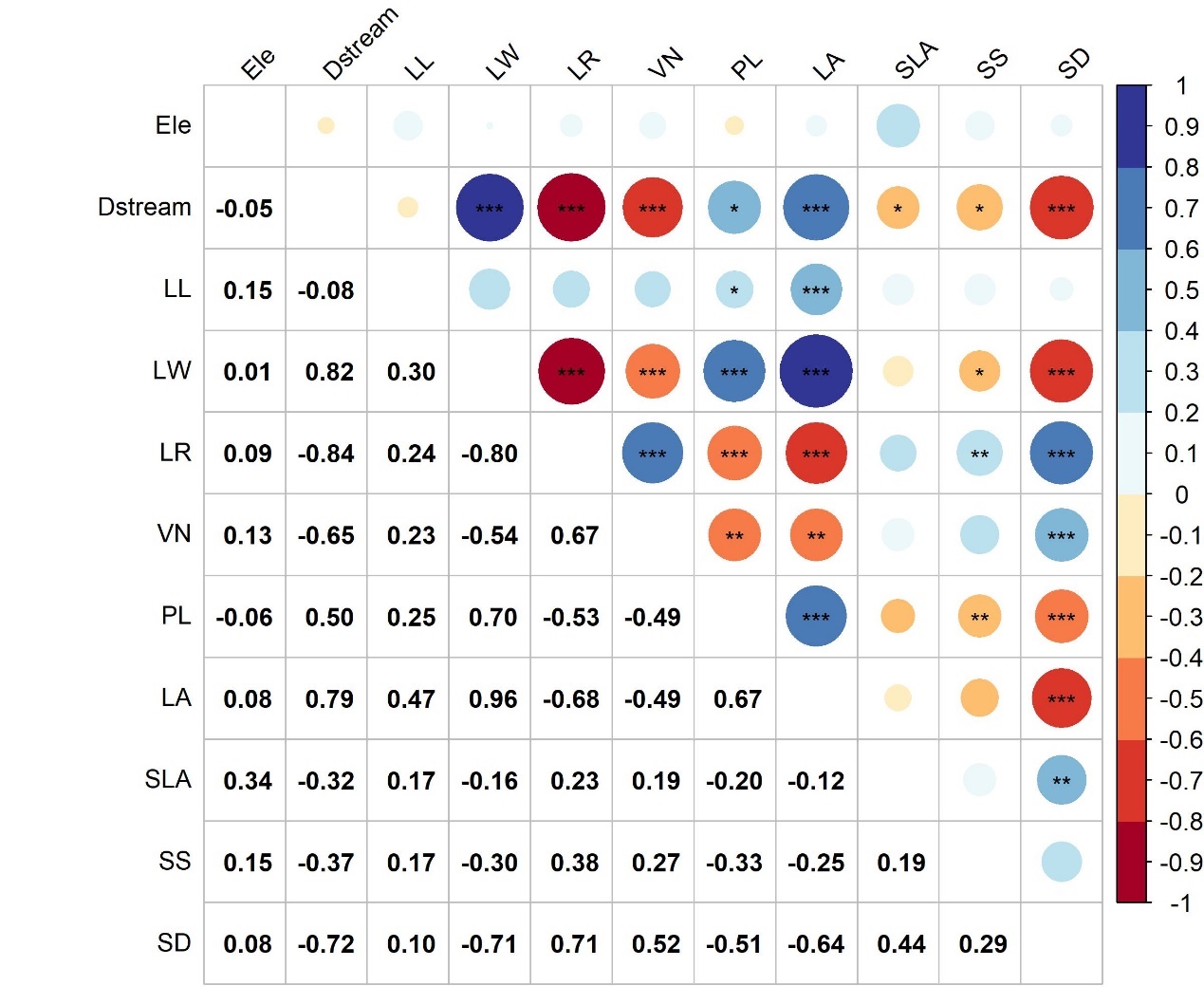


**Figure S5**. Spearman’s rank correlation coefficients (lower-left) for all pairwise trait comparisons using pooled data from 43 individuals of *Ficus* *hispida*, *F. squamosa*, and putative hybrids. Significant result for coefficients (upper-right); *p*<0.001, ***; *p*<0.01, **; *p*<0.05, *. Trait abbreviations: Ele, Elevation; Dstream, Distance from stream; LL, Leaf length; LW, Leaf width; LR, Leaf ratio; VN, Vein count; PL, Petiole length; LA, Leaf area; SLA, Specific leaf area; SS, Stomatal size; SD, Stomatal density.

**Germination Test**

Seeds of *F. hispida*, *F. squamosa*, and putative hybrids were tested for germination, with 30 seeds per individual. Due to the small sample size, these results may not be conclusive. However, one of the putative hybrids demonstrated faster germination (10.09 days), a higher number of germinated seeds, a higher germination percentage (80%), and a greater germination index (2.34) (**Figure S7**). This suggests that putative hybrids may possess enhanced germination traits, potentially indicating hybrid vigor or adaptive advantages in certain environmental conditions. Further studies with larger sample sizes are needed to confirm these findings and understand the ecological implications of hybridization in *Ficus* species.


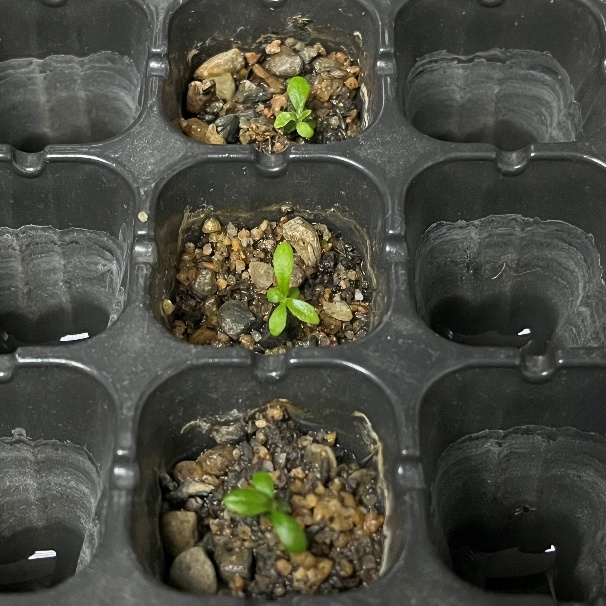

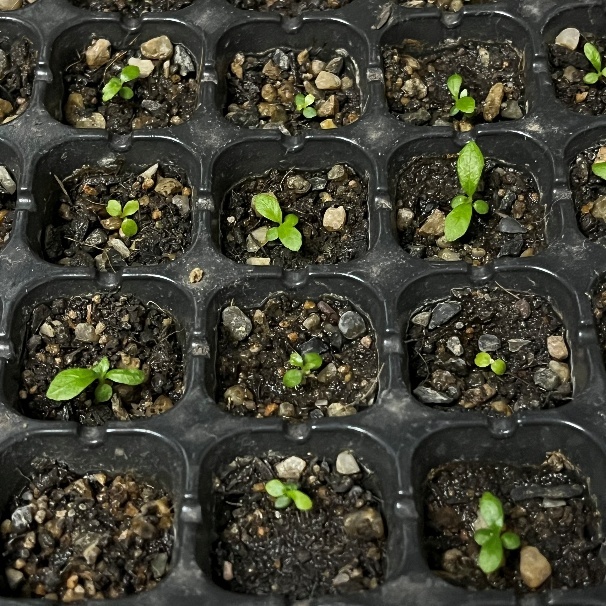


**Figure S6**. Example seedling of *F. squamosa* (left) and putative hybrid (right).


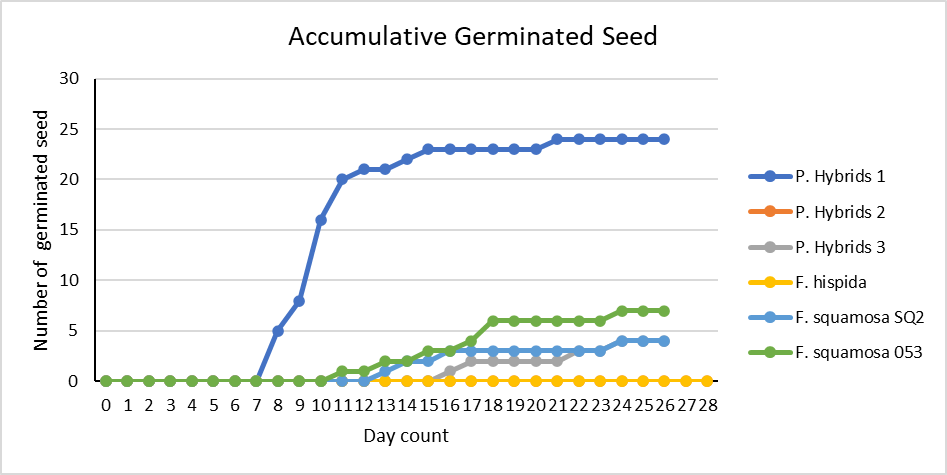


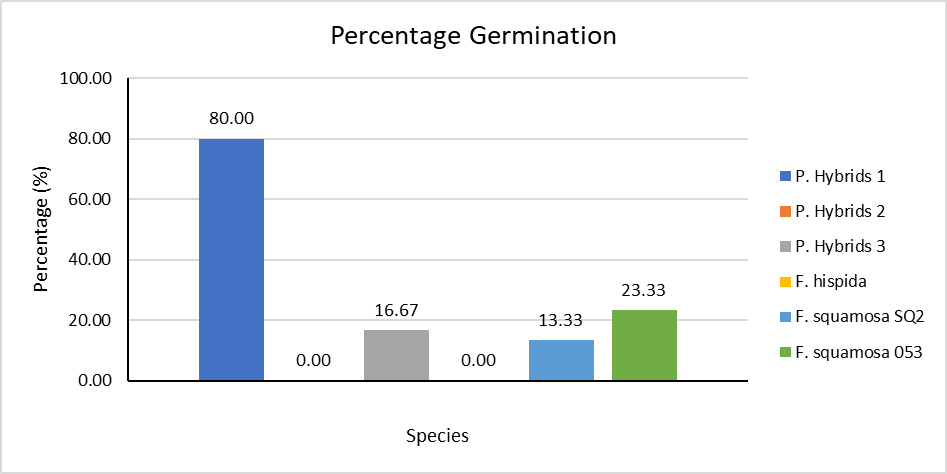


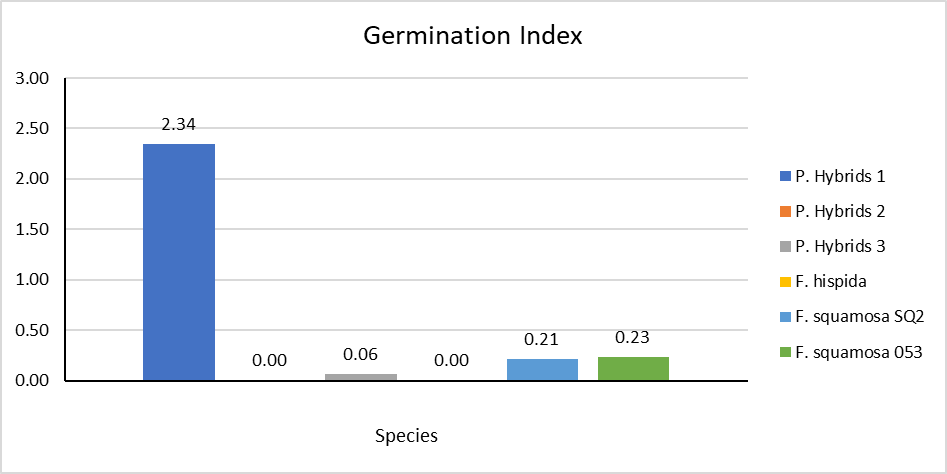


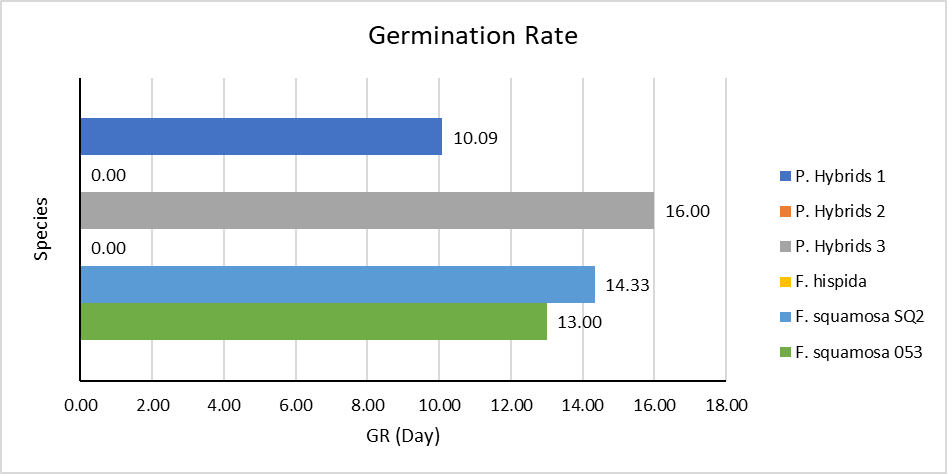


**Figure S7**. Detail on next page.

**Figure S7**. Accumulative germinated seed, percentage germination, germination index, and germination rate of the three taxa (putative hybrids, *F. hispida*, and *F. squamosa*)

**Fig Wasp Characteristics**

The comparative of morphological characteristics between two species of fig wasps, *F. hispida* and *F. squamosa*. Both wasps have an antennal flagellum with eight segments and a yellowish-brown coloration. However, while both species have eleven antennal segments in total, the *F. squamosa* wasp's antennal flagellum is slightly narrower and longer. The scape is yellowish-brown in both species, while the head is dark brown in both. A key difference is observed in the mesosoma: it is dark brown in *F. hispida* but yellowish in *F. squamosa*. The metasoma is consistently dark brown in both species. The mandibles are yellowish-brown in both wasps, and their legs are yellowish. This comparison subtle morphological variations between the two wasp species, which could be adaptations to their respective host fig species (**Figure S9-S14**).

Hybridization in fig wasps is a very rare phenomenon, as these insects typically exhibit strong host specificity, which limits opportunities for cross-species encounters. However, hybridization remains uncertain, especially between closely related species like *F. hispida* and *F. squamosa*, whose hosts—*F. hispida*, *F. squamosa*, and *F. heterostyla*—are closely related. This suggests that these wasp species may share morphological and genetic compatibility, facilitating occasional hybridization. Interestingly, in *F. squamosa* and its hybrids, both light-colored and dark-colored wasps have been observed in Chiang Mai, Thailand (RK and PP personal observation). Additionally, in a hybrid tree, two fig wasp morphs—both light and dark—were found inhabiting the same tree but occupying different figs. This observation raises intriguing questions about the ecological and genetic factors influencing wasp distribution and hybridization, suggesting a complex interaction between host specificity, genetic variation, and environmental influences.

Further investigation using light microscopy and morphometric analysis revealed no significant differences between *F. hispida* and *F. squamosa* in key characteristics such as head length, thorax length, abdomen length, total body length, and ovipositor length. However, *F. squamosa* wasps were found to have a significantly narrower head width. This suggests that *F. squamosa* wasps are capable of passing through the ostiole of *F. hispida* figs. Additionally, the lack of a significant difference in ovipositor length indicates that *F. squamosa* wasps can lay their eggs in the gall flowers of *F. hispida*, further supporting the possibility of cross-host utilization and potential hybridization between these closely related wasp species (**Figure S8**).


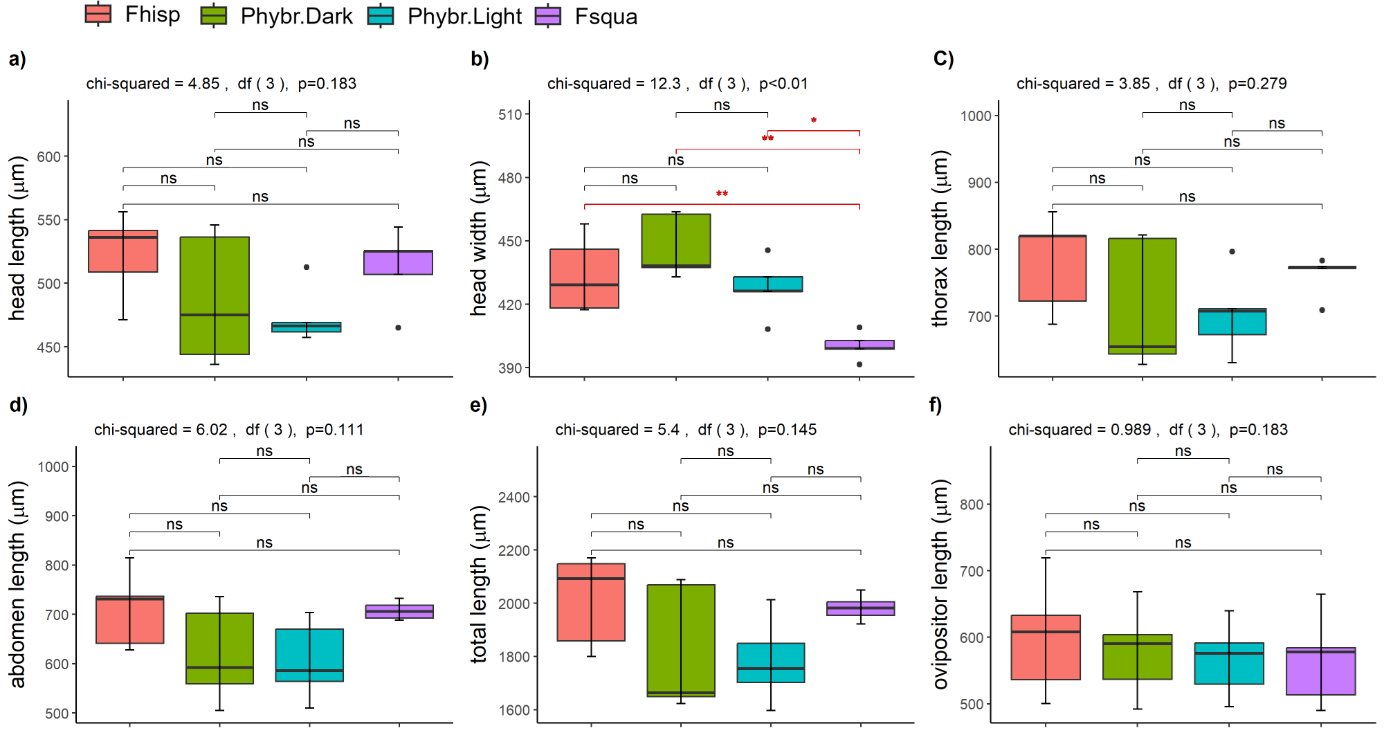


**Figure S8**. Box plot showing the results of the Kruskal-Wallis test for wasp characteristics, including (a) head length, (b) head width, (c) thorax length, (d) abdomen length, (e) total length, and (f) ovipositor length. *F. hispida* wasps are represented in pinkish-red, dark putative hybrid wasps in green, light putative hybrid wasps in cyan blue, and *F. squamosa* wasps in pastel purple. Statistical significance is indicated as p < 0.01 (**), p < 0.05 (*), and "ns" for no significant difference**.**


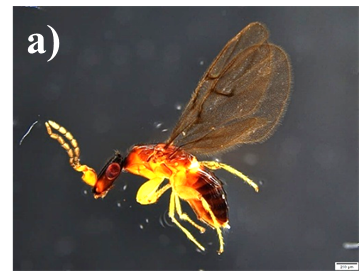

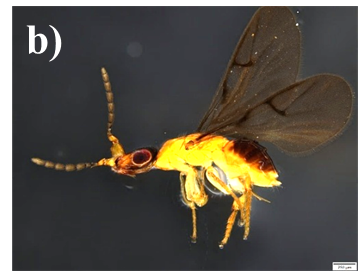


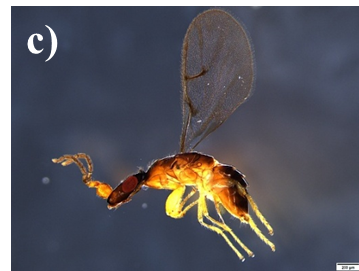

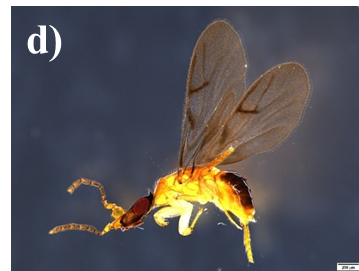


**Figure S9**. Whole Body of a) *F. hispida*, b) *F. squamosa*, c) putative hybrid-dark, and d) putative hybrid-light wasps.


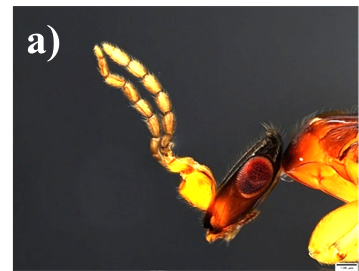

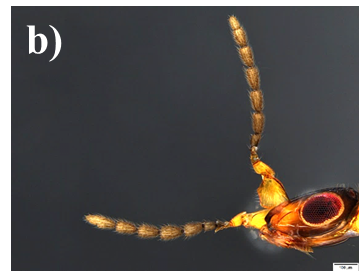


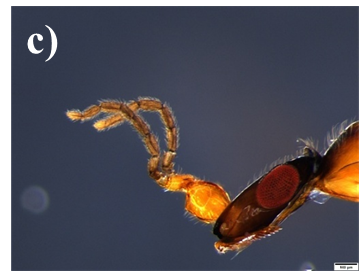

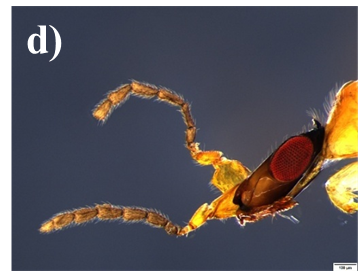


**Figure S10**. Antenna of a) *F. hispida*, b) *F. squamosa*, c) putative hybrid-dark, and d) putative hybrid-light wasps**.**


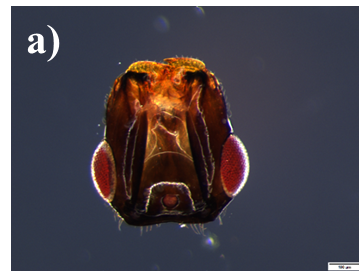

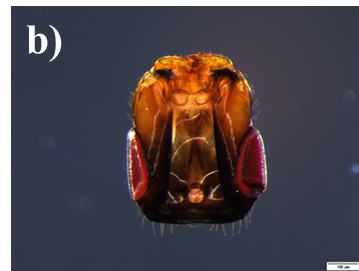


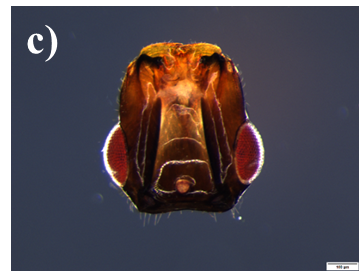

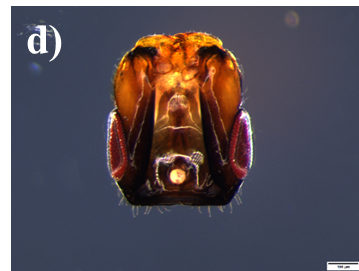


**Figure S11**. Head (dorsal) of a) *F. hispida*, b) *F. squamosa*, c) putative hybrid-dark, and d) putative hybrid-light wasps.


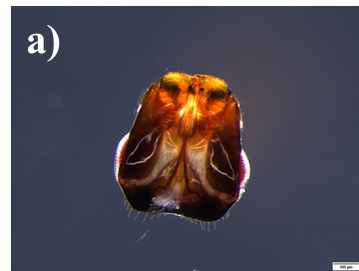

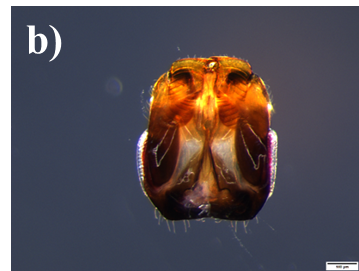


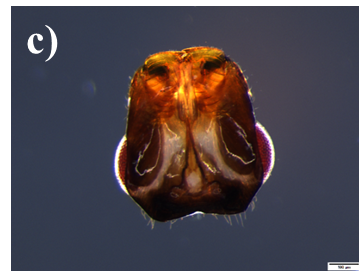

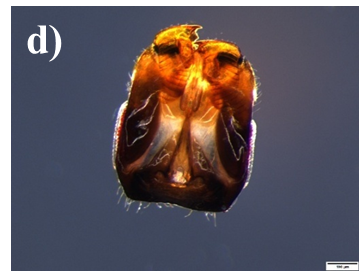


**Figure S12**. Head (ventral) of a) *F. hispida*, b) *F. squamosa*, c) putative hybrid-dark, and d) putative hybrid-light wasps**.**


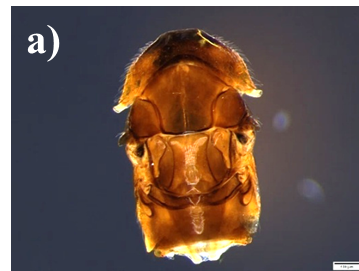

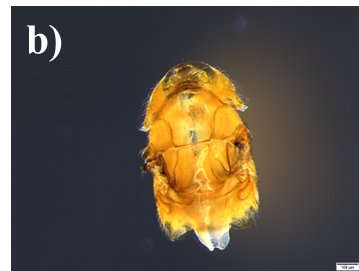


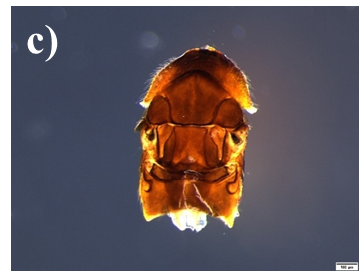

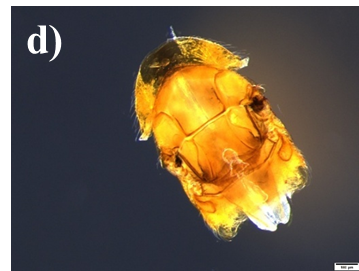


**Figure S13**. Thorax (dorsal) of a) *F. hispida*, b) *F. squamosa*, c) putative hybrid-dark, and d) putative hybrid-light wasps.


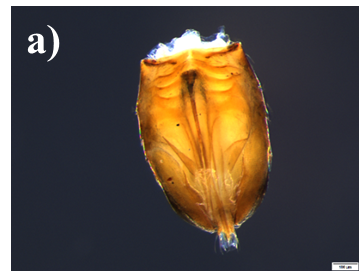

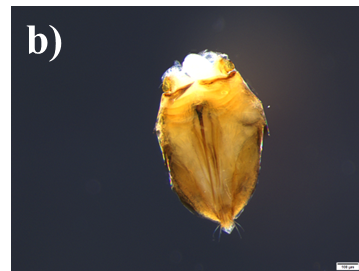


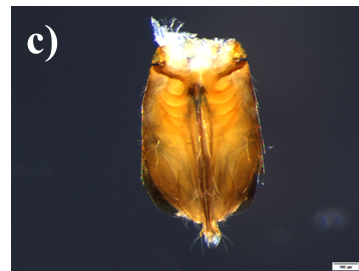

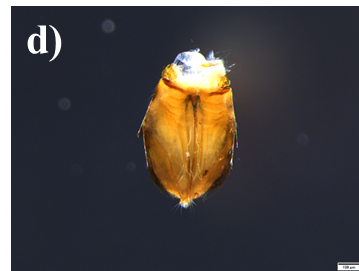


**Figure S14**. Abdomen (ventral, ovipositor) of a) *F. hispida*, b) *F. squamosa*, c) putative hybrid-dark, and d) putative hybrid-light wasps.
